# Pharmaceutical company spending on research and development and promotion in Canada, 2013-2016: a cohort analysis

**DOI:** 10.1186/s40545-018-0132-3

**Published:** 2018-03-13

**Authors:** Joel Lexchin

**Affiliations:** 1 School of Health Policy and Management, York University, 4700 Keele St., Toronto, ON M3J 1P3 Canada; 2 University Health Network, 200 Elizabeth St., Toronto, ON M5G 2C4 Canada

**Keywords:** Canada, Expenditure, Pharmaceutical industry, Promotion, Research and development

## Abstract

**Background:**

Competing claims are made about the amount of money that pharmaceutical companies spend on research and development (R&D) versus promotion. This study investigates this question in the Canadian context.

**Methods:**

Two methods for determining industry-wide figures for spending on promotion were employed. First, total industry spending on detailing and journal advertising for 2013-2016 was abstracted from reports from QuintilesIMS. Second, the mean total promotion spending for the years 2002-2005 was used to estimate total spending for 2013-2016. Total industry spending on R&D came from the Patented Medicine Prices Review Board (PMPRB). R&D to promotion spending using each method of determining the amount spent on promotion was compared for 2013-2016 inclusive. Data on the 50 top promoted drugs, the amounts spent, the companies marketing these products and their overall sales were abstracted from the QuintilesIMS reports. Spending on R&D and promotion as a percent of sales was compared for these companies.

**Results:**

Industry wide, the ratio of R&D to promotion spending went from 1.43 to 2.18 when promotion was defined as the amount spent on detailing and journal advertising for the 50 most promoted drugs. Calculating total promotion spending from the mean of the 2002-2005 figures the ratio was 0.88 to 1.32 for the 50 most promoted drugs. For individual companies marketing one or more of the 50 most promoted drugs, mean R&D spending ranged from 3.7% of sales to 4.1% compared to mean promotion spending that went from 1.7 to 1.9%. The ratio of spending on R&D to promotion varied from 2.11 to 2.32. Eight to 10 companies per year spent more on promotion than on R&D.

**Conclusions:**

Depending on the method used to determine promotion spending, industry-wide the ratio of R&D spending to promotion ranges from 1.45 to 2.18 (sales representatives and journal advertising only) or from 0.88 to 1.32 (total promotion spending estimated based 2003-2005 data.) For the individual companies promoting one or more of the 50 most promoted drugs, 2.11 to 2.32 times more is spent on R&D compared to promotion. However these results should be interpreted cautiously because of data limitations.

**Electronic supplementary material:**

The online version of this article (10.1186/s40545-018-0132-3) contains supplementary material, which is available to authorized users.

## Background

Pharmaceutical companies typically claim that one of the reasons for high drugs prices is because of the amount that they spend on research and development (R&D). According to the industry, it costs USD $2.6 billion to bring a drug to market [1]. Critics of the industry counter that companies are more focused on and spend more on promotion than on R&D. Gagnon and Lexchin produced figures that showed that in the United States (US) in 2004 the industry spent USD $57.5 billion on promotion versus USD $31.5 billion on R&D [2]. A report from the California-based Institute for Health and Socio-Economic Policy stated that in 2015 out of the top 100 pharmaceutical companies by sales, 64 spent twice as much on marketing and sales than on R&D, 58 spent three times, 43 spent five times as much and 27 spent 10 times the amount [3]. To date, arguments about promotion versus R&D spending have been based on American data. This study had two aims: first to estimate total industry-wide promotion and compare that to total industry-wide R&D spending and second, to look at the ratio of R&D versus promotional spending for individual companies marketing the most heavily promoted drugs in Canada.

## Methods

### Industry-wide

Two methods were used to determine industry-wide promotion spending. First, total spending on sales representatives and journal advertising, i.e., excluding all other types of promotion, for the top 50 most heavily promoted products from companies was available for the years 2013-2016 from the annual reports published by QuintilesIMS (formerly imshealth|brogan) [4–7].

Second, amounts for all types of promotion in Canada were available for the period 2002-2005 from Cammcorp International (Marc-Andre Gagnon, personal communication, January 15, 2011). The mean ratio of spending on detailing plus journal advertising to total promotion spending was calculated for that period and that ratio was then used to convert annual spending on detailing and journal advertising to total promotion spending for the years 2013-2016, assuming that the relative proportions spent on detailing plus advertising were the same in 2013-2016 as they were in 2002-2005. These calculated amounts constitute a floor for total promotional spending since the figures for 2013-2016 only cover the top 50 most promoted products. As an illustrative example, between 2002 and 2005 detailing plus advertising was a mean of 60.4% of total industry-wide promotional spending of $1.066 billion. In 2013, spending on detailing plus advertising for the 50 most heavily promoted drugs was $0.575 billion and therefore the calculated lower total amount was 0.575/60.4 × 100 = $0.952 billion. The calculation was repeated for the years 2014-2016.

Total R&D spending by all companies with a patented drug on the Canadian market for the same 4-year period was available from the annual reports from the Patented Medicine Prices Review Board (PMPRB), the federal body that sets a maximum introductory price for new patented medicines [8–11]. Ratios of spending on R&D to promotion were calculated for each year using the two different methods of determining annual promotion spending.

### Companies with the most heavily promoted drugs

The names of the 50 medications with the highest spending on promotion, the companies marketing these medications and the amount spent were abstracted for the years 2013 to 2016 inclusive from QuintilesIMS annual reports. These reports also contain total sales revenue for the top 50 companies in Canada and these figures along with figures on promotion spending was entered into an Excel spreadsheet. The amount spent on promotion by the top companies was summed and the mean percent of sales spent on promotion was also calculated for all of the top companies for each of the 4 years.

The percent of sales spent by companies on R&D was abstracted from the annual PMPRB reports for each of the companies with the most heavily promoted drugs and the amount spent on R&D for each company was calculated by multiplying this figure by the total sales of each company found in the QuintilesIMS reports. The combined amount and the mean percent of sales spent on R&D by the companies was calculated for each year.

Combined R&D spending and the mean percent of sales spent on R&D was compared to similar figures for promotion for the companies with the most heavily promoted drugs.

All of the data was publicly available and no patients were involved. Therefore, ethics approval was not necessary.

## Results

### Industry-wide

Total industry R&D spending, where industry is defined as all companies with a patented drug on the Canadian market, ranged from a low of $792,200,000 to a high of $918,200,000. Spending on sales representatives and journal advertising for the 50 most heavily promoted drugs went from a low of $421,434,000 to $562,926,000 and the ratio of R&D to promotion spending was 1.43 to 2.18. Total promotion spending on the 50 most heavily promoted drugs, calculated from the mean of the 2002-2005 figures, ranged from $697,000,000 to $932,000,000 and the ratio was 0.88 to 1.32 (Table 1).

**Table 1:** Spending on R&D^a^ and promotion as a percent of sales industry-wide

Year	Total R&D spending ($000)	Promotion spending ($000)		Ratio R&D to promotion spending	
		Detailing and journal advertising top 50 drugs	Estimated total promotion based on data from 2002-2005	Promotion for top 50 drugs	Estimated total promotion
2013	798,300	550,871	912,000	1.45	0.88
2014	792,200	552,797	916,000	1.43	0.86
2015	869,100	562,926	932,000	1.54	0.93
2016	918,200	421,434	697,000	2.18	1.32

^a^Research and development

### Companies with the most heavily promoted drugs

In each of the 4 years, data was not available for a small number of the 50 most promoted drugs: 2013 – 4 drugs (no total sales figures), 2014 and 2015 – 4 drugs (no total sales figures) and 1 drug (no R&D as a percent of sales), 2016 – 1 drug (no total sales figure). (Complete data on individual companies for each year is available in Additional file 1: Table S1, Additional file 2: Table S2, Additional file 3: Table S3, Additional file 4: Table S4.)

R&D and promotion spending was available for 22 companies in each of the 4 years. (The individual companies selling the top 50 drugs varied from year to year and overall 26 unique companies had data for 1 to 4 years.) In 2016, the amount spent on promotion for one company included company promotion rather than promotion for an individual product. For the majority of companies (15-17 out of 22 in any given year) the amount spent on promotion was only available for 1 or 2 drugs (Table 2). Mean R&D spending ranged from 3.7% of sales to 4.1% compared to mean promotion spending that went from 1.7 to 1.9%. The ratio of spending on R&D to promotion varied from 2.11 to 2.32 (Table 3). Depending on the year, between 8 to 10 of the 22 companies spent more on promotion than on R&D (Table 3).

**Table 2:** Number of 50 most promoted drugs per company per year

Year	Number of drugs promoted						
	1	2	3	4	5	6	7
2013	10	5	3	3	1		
2014	10	6	3	2	1		
2015	10	7	1	2	2		
2016	10	6	2	2		1	1

**Table 3:** Spending on R&D^a^ and promotion as a percent of sales for companies with the most heavily promoted drugs

Year	Number of companies^b^	Number of drugs	Total sales ($000)	R&D spending ($000) (% sales)	Promotion spending ($000) (% sales)	Ratio of R&D to promotion spending	Number of companies spending more on promotion than on R&D
2013	22	46	12,761,798	565,670 (4.4)	243,420 (1.9)	2.32	9
2014	22	46	13,327,819	509,531 (3.8)	242,401 (1.8)	2.11	8
2015	22	45	13,215,385	487,223 (3.7)	230,157 (1.7)	2.18	9
2016	22	49	13,610,805	553,057 (4.1)	258,363 (1.9)	2.16	10

^a^Research and development

^b^Individual companies vary by year

**Fig. 1:**
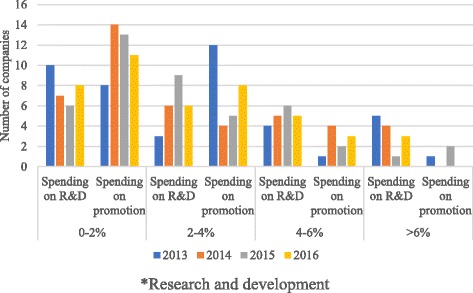
Percent of sales spend on R&D* and promotion by number of companies. *Research and development

Figure 1 shows the distribution of spending on R&D versus promotion as a percent of sales by number of companies. Companies tended to cluster at the lower end for spending on promotion whereas they were at the higher end for spending on R&D. The largest amount spent by an individual company on R&D was 13.6% in 2016 and on promotion it was 7.8% in 2015. Table 4 shows that for most companies there was significant variation in spending on both promotion and R&D on a year-to-year basis.

**Table 4:** Variation in promotion and research and development spending by individual company by year

Company	Promotion spending on drugs in 50 most promoted ($000)				Research and development spending ($000)			
	2013	2014	2015	2016	2013	2014	2015	2015
Abbott EPD	15,779	16,009	8104	–	0	0	0	–
Actavis	–	3034	2753	–	–	20	0	–
Allergan	–	–	–	2328	–	–	–	1751
Amgen	5816	5836	6515	6359	50,370	45,622	42,153	38,600
Astellas	3006	3097	8094	5612	4735	3447	6952	3851
AstraZeneca	20,066	35,416	33,655	41,304	13,781	24,273	41,143	61,468
Bayer	10,857	11,995	11,486	11,898	23,476	31,373	39,719	45,025
BGP Pharma	–	–	–	4809	–	–	–	0
BMS Pharma	20,081	13,132	7588	14,741	53,270	45,001	59,456	81,335
Boehringer Ingelheim	11,570	17,304	14,147	24,961	23,476	18,430	22,742	21,409
GlaxoSmithKline	19,384	30,547	13,328	27,567	88,350	77,648	53,413	43,326
Janssen	5636	12,666	16,839	6356	49,983	61,733	70,646	86,380
Leo	3847	–	–	–	939	–	–	–
Lilly	12,358	8261	7592	6150	53,672	26,620	22,277	36,710
Lundbeck	9956	3322	12,146	8900	888	2596	3126	0
Merck	27,682	13,065	18,441	36,963	19,962	26,505	21,667	32,582
Novartis	3460	7838	5376	12,002	106,013	78,553	51,916	44,701
Novo Nordisk	6536	4159	3517	3580	4563	10,857	7886	4548
Paladin	–	–	3113	–	–	–	265	–
Pfizer	24,429	12,009	12,178	14,162	24,643	14,174	8953	9738
Purdue	9996	3790	6099	2666	13,581	14,683	12,609	16,321
Sanofi-Aventis	3222	2870	–	3578	19,940	13,847	–	8450
Servier	7862	14,134	8893	7252	11.745	13,612	8067	7150
Shire	4145	6967	3287	3464	281	350	0	0
Takeda	14,099	13,415	19,582	5460	0	0	0	0
Valeant	3633	3535	7414	8251	0	0	14,231	9712

## Discussion

Depending on the method used to determine promotion spending, industry-wide the ratio of R&D spending to promotion ranges from 1.45 to 2.18 (sales representatives and journal advertising only) or from 0.88 to 1.32 (total promotional spending) for the years 2013 to 2016. The amount spent on promotion used in calculating the former set of ratios is more accurate but is limited to just two types of promotion. The amount spent on promotion used in calculating the latter set of ratios is an estimate for all types of promotion but is less accurate. Both sets of ratios also only apply to the amount spent on the 50 most heavily promoted drugs. For the individual companies promoting one or more of the 50 most promoted drugs, 2.11 to 2.32 times more is spent on R&D compared to promotion. Even using the limited data on individual company promotion, 8-10 companies per year still spent less on R&D than they did on promotion.

However, all of these results should be interpreted cautiously for a number of reasons. First, the QuintilesIMS reports only provide figures for spending on sales representatives and journal advertising whereas companies engage in a number of other methods of promotion, among them, distributing free samples, hiring key opinion leaders to give talks, sponsoring meals and meetings and using social media. Second, the estimate for total promotional spending based on the mean of the 2002-2005 figures assumes that the proportion of the total used on journal advertising and detailing remained stable. Finally, for many companies promotion spending was based on the amount spent on just 1 or 2 drugs.

The reason for the abrupt decline in spending on detailing and journal advertising in 2016 compared to 2013-2015 requires further investigation. The variation in the year-to-year amount spent on both R&D and promotion by individual companies most likely reflects research priorities and how many drugs companies are aggressively marketing.

Beyond examining the amount of money spent on R&D and promotion, looking at the focus of that spending and the outcomes is another way of assessing the priorities that companies attach to each of these activities. Innovative Medicines Canada (IMC), the organization representing the research-based industry, signals the importance that it attaches to R&D on its website: “At present, there are more than 500 new products in development in Canada, including therapies focused on cancer treatments, infectious diseases and vaccines. These products have the potential to help Canadians and people all over the world live longer and healthier lives” [12]. However, industry spending on R&D in Canada has been declining ever since 1997/1998 when it peaked at 11.5% of sales and it now stands at 4.4% [11]. Out of 564 new patented medicines introduced into Canada from 2010 to 2016 inclusive, only 37 (6.6%) were rated by the PMPRB as breakthroughs or substantial therapeutic improvements [11]. Although most products that fail in the clinical development stage do so because of a lack of efficacy or for safety reasons, 57% and 17% respectively, 22% are stopped for commercial reasons [13] indicating that products that might be both safe and efficacious are not brought to market.

Industry is also positive about the role of promotion, especially the activities of sales representatives. This orientation is reflected in a statement about the role of pharmaceutical sales representatives issued by Rx&D, the predecessor to IMC: “Provider-supported detailing generates awareness about new treatments and provides science-based and Health Canada approved advice on how to administer these medications” [14]. Previous research that examined the most heavily promoted products in Canada, showed that the vast majority of spending went to medications that offered little to no additional therapeutic value over existing therapies [15]. The comprehensiveness of the safety information provided by sales representatives when they visit doctors was investigated in a study involving primary care practitioners in Vancouver and Montreal. “Minimally adequate safety information” defined a priori as the mention of one or more of the following: approved indications, serious adverse events, common non-serious adverse events and contraindications *and* no unapproved indications or unqualified safety claims (e.g., “this drug is safe”) was provided in 5/412 (1.2%) of promotions in Vancouver and 7/423 (1.7%) in Montreal. Representatives did not provide any information about harms (a serious adverse event, a common adverse event or a contraindication) in two-thirds of interactions [16].

It would appear that regardless of the amount of money spent on these two activities – R&D or promotion – that commercial objectives are one of the main considerations behind how pharmaceutical companies direct their expenditures.

### Limitations

These results only apply to companies that market the top 50 most promoted drugs in Canada in any given year. Results for other companies may vary. The pharmaceutical industry also disputes the R&D figures in the annual PMPRB reports because these figures are based on the definition of R&D used by Revenue Canada, whereas according to the industry other types of investment should also be included, increasing the amount of R&D spending [17].

## Conclusion

Based on the available data, individual pharmaceutical companies in Canada, on average, are spending more on R&D than on promotion; however, the reverse is true for a minority of companies. For the industry as a whole, more may be spent on promotion versus R&D although a definite conclusion would require access to more complete data.

## Additional files

Additional file 1: **Table S1.** Company spending on R&D and promotion – 2013. (XLSX 10 kb)

Additional file 2: **Table S2.** Company spending on R&D and promotion – 2014. (XLSX 10 kb)

Additional file 3: **Table S3.** Company spending on R&D and promotion – 2015. (XLSX 9 kb)

Additional file 4: **Table S4.** Company spending on R&D and promotion – 2016. (XLSX 10 kb)

## Abbreviations

IMC: Innovative Medicines Canada
PMPRB: Patented Medicine Prices Review Board
R&D: Research and development
